# Dominance of the residential sector in Chinese black carbon emissions as identified from downwind atmospheric observations during the COVID-19 pandemic

**DOI:** 10.1038/s41598-021-02518-2

**Published:** 2021-12-16

**Authors:** Yugo Kanaya, Kazuyo Yamaji, Takuma Miyakawa, Fumikazu Taketani, Chunmao Zhu, Yongjoo Choi, Kohei Ikeda, Hiroshi Tanimoto, Daichi Yamada, Daiju Narita, Yutaka Kondo, Zbigniew Klimont

**Affiliations:** 1 Graduate School of Maritime Sciences, Kobe University, Kobe, 6580002 Japan; 2 Research Institute for Global Change, Japan Agency for Marine-Earth Science and Technology (JAMSTEC), Yokohama, 2360001 Japan; 3 Earth System Division, National Institute for Environmental Studies, Tsukuba, 3058506 Japan; 4 Faculty of Economics and Business, Hokkaido University, Sapporo, 0600809 Japan; 5 The University of Tokyo, Tokyo, 1538902 Japan; 6 National Institute of Polar Research, Tachikawa, 1908518 Japan; 7 International Institute for Applied Systems Analysis (IIASA), 2361 Laxenburg, Austria

**Keywords:** Atmospheric chemistry, Atmospheric chemistry, Climate-change mitigation

## Abstract

Emissions of black carbon (BC) particles from anthropogenic and natural sources contribute to climate change and human health impacts. Therefore, they need to be accurately quantified to develop an effective mitigation strategy. Although the spread of the emission flux estimates for China have recently narrowed under the constraints of atmospheric observations, consensus has not been reached regarding the dominant emission sector. Here, we quantified the contribution of the residential sector, as 64% (44–82%) in 2019, using the response of the observed atmospheric concentration in the outflowing air during Feb–Mar 2020, with the prevalence of the COVID-19 pandemic and restricted human activities over China. In detail, the BC emission fluxes, estimated after removing effects from meteorological variability, dropped only slightly (− 18%) during Feb–Mar 2020 from the levels in the previous year for selected air masses of Chinese origin, suggesting the contributions from the transport and industry sectors (36%) were smaller than the rest from the residential sector (64%). Carbon monoxide (CO) behaved differently, with larger emission reductions (− 35%) in the period Feb–Mar 2020, suggesting dominance of non-residential (i.e., transport and industry) sectors, which contributed 70% (48–100%) emission during 2019. The estimated BC/CO emission ratio for these sectors will help to further constrain bottom-up emission inventories. We comprehensively provide a clear scientific evidence supporting mitigation policies targeting reduction in residential BC emissions from China by demonstrating the economic feasibility using marginal abatement cost curves.

## Introduction

Air pollutant emissions from human activities have driven major changes in the atmospheric composition, exerting climate change and human health impacts^1–4^. The mitigation of these issues typically relates to emission reductions and therefore, accurate quantification and estimation of the current total emission fluxes, sector contributions, geographical distributions, and their temporal changes are crucial for efficient and robust mitigation policies. The linkage between temporal changes in the emission fluxes and atmospheric concentrations also needs to be understood to clarify the causal relationships.

Bottom-up emission inventories based on socio-economic information contain all such necessary information. However, large uncertainties are associated with the reported emission rates, by up to a factor of two for of black carbon (BC) particles^5,6^, arising from uncertainties of the emission factors and activity data. The uncertainty might be reduced when constrained with atmospheric concentration observations, i.e., where the numerical model simulations driven with the emission inventory are compared with observations. For example, long-term satellite-based and high-quality ground-based observations have provided trend information from which the relevant emission trends could be inferred^7,8^. In some advanced analyses, caution is exercised to account for the non-linear relationship between the emissions and concentrations owing to atmospheric chemistry as well as the effects from confounding factors such as changes in the air flow patterns or in the wet deposition^9,10^. However, a major limitation of this approach is that sector-specific evaluation is typically unattainable, unless the analysis is combined with good tracers that are specific for a particular sector. For example, in our previous work, total BC emission fluxes from China and their trend during 2009–2019 have been successfully estimated from atmospheric BC concentration observations at Fukue Island, located downwind of China with respect to the winter monsoon^10^; however, the contributions from industry and transport and those from the residential sectors were difficult to separate from each other.

Restriction in the human activity due to the prevalence of COVID-19 in spring 2020 resulted in sharp decrease of activity levels for certain sectors, especially transport and industry, while the residential sector activity remained essentially unchanged, providing a unique opportunity to separate the contributions.

In this study, we exemplify such separation for the first time for BC and carbon monoxide (CO) emission fluxes of Chinese origin, from the analysis of long-term observations at Fukue Island, western Japan, in February and March, during 2015–2020. The analysis was aided by atmospheric chemistry transport model simulations, which were used to remove the confounding factors from meteorology. The reduced emissions in 2020, due to the COVID-19 outbreak and compared with 2019 level, were used to infer the fractional contributions from the transport and industrial sectors. First, considering available data and information about the extent of the reduced activity levels, the full emission flux from the non-residential sectors was estimated. Then the remaining emission flux was attributed to the residential sector, which was assumed as unchanged relative to 2019 levels^11^. Policy implication of the results is also discussed.

## Results and discussion

### Estimating sector-specific emission fluxes

**Figure 1:**
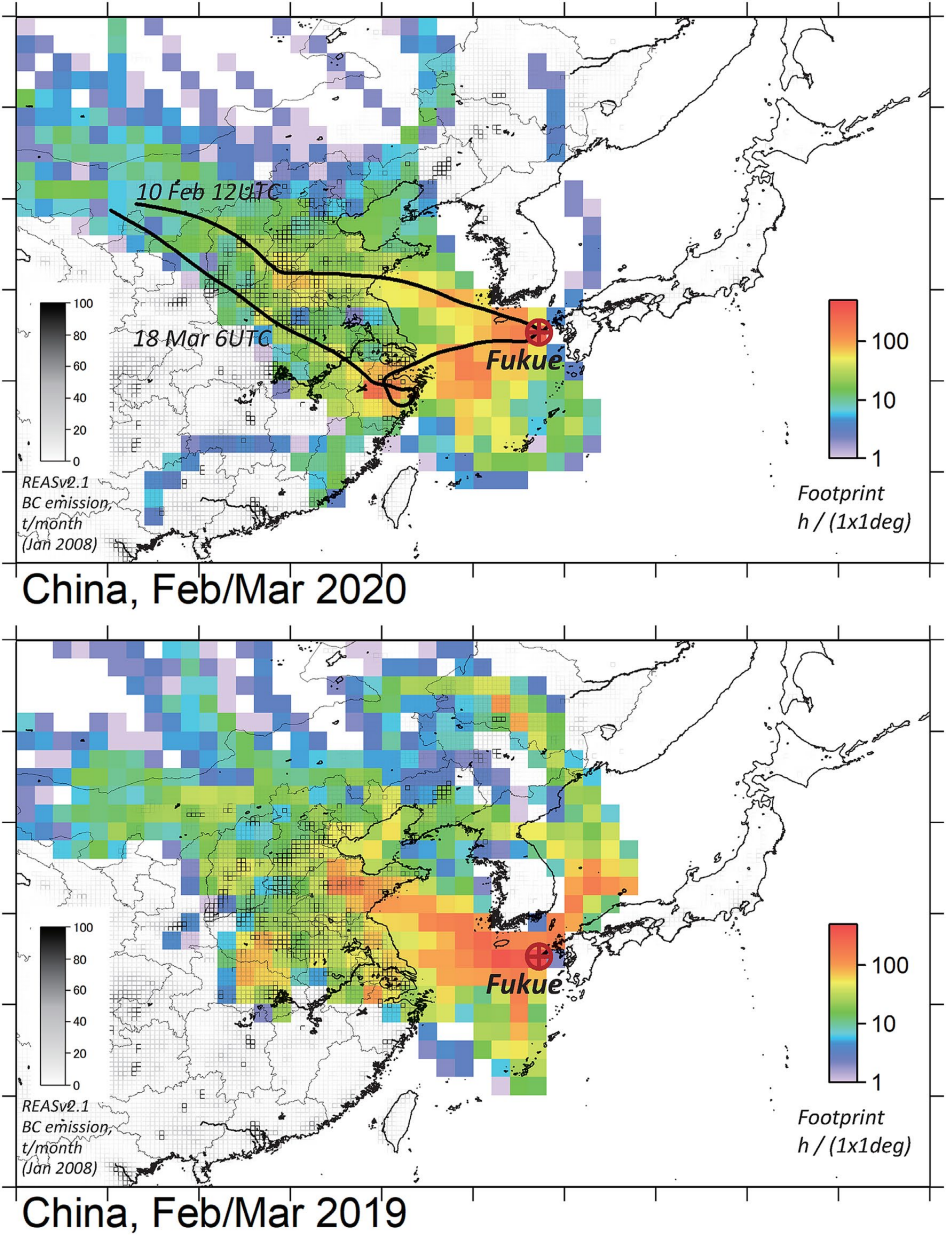
Location of Fukue atmospheric environment observatory and footprint density distribution during (top) Feb–Mar 2020 and (bottom) Feb–Mar 2019. For 2020, two backward trajectories are also depicted. BC emission distribution from REAS version 2.1^16^ is indicated with squares with gray-scaled borderlines.

During Feb–Mar 2020, when the strongest reduction in the Chinese emission flux was estimated^12^, air masses reached Fukue Island (32.75°N, 128.68°E, 75 m above sea level, Fig. 1, see “Methods”) from major economic centers over China in the 108 hourly cases according to the 120-h backward trajectories (using HYSPLIT^13^, see “Methods”), with negligible loss of BC by the wet removal process during travel, as assessed using the accumulated precipitation along the trajectory (APT, see “Methods”) as an index^10,14^. The backward trajectory calculations yielded 4215 hourly waypoints over China (at altitudes below 2500 m) in total, suggesting a footprint distribution covering a wide area of central East China (CEC) with large BC emission rates (Fig. 1, top); for example at 10 Feb 1200 UTC and 18 Mar 0600 UTC, backward trajectories passed over the north and south parts of CEC, respectively. The geographical pattern of the footprint density was similar to that from Feb–Mar 2019, estimated with 6230 hourly waypoints over China (Fig. 1, bottom), and also to those from earlier years during 2015–2018 (Fig. S1). Note that the footprint calculated with the FLEXPART model^15^ considering dispersion (see “Methods”) had a relatively wider coverage but showed similar spatial patterns (Fig. S2).

**Figure 2:**
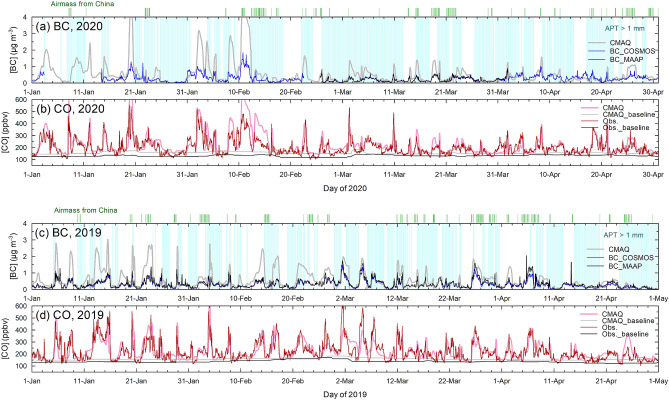
Time series of (**a**) BC mass concentrations and (**b**) CO mixing ratios from observations (dark colors) and model simulations (light colors) during Jan–Apr in 2020 and 2019 (**c**, **d**). The estimated baseline levels are also shown. The hours with arrival of air masses from China are indicated with green vertical bars. Note that the cases from China but via Korea are not included to avoid false assignment. Light blue lines indicate cases with non-negligible wet deposition.

**Figure 3:**
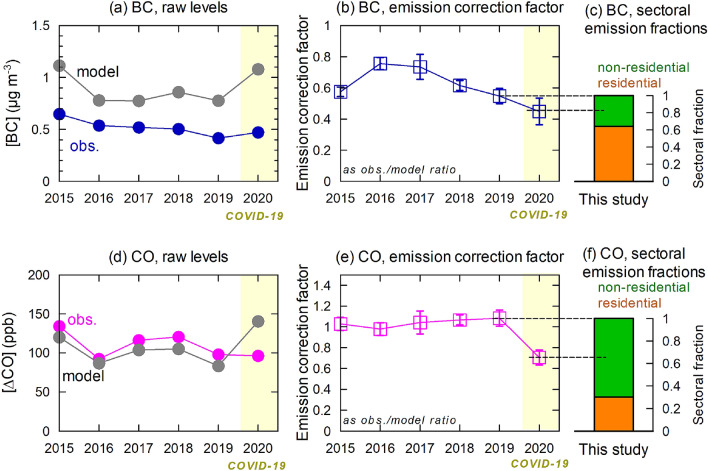
(**a**) Observed and modeled BC mass concentration levels for selected air masses (from China) during Feb–Mar for each year, (**b**) their ratios for estimating emission correction factors, and (**c**) estimated fractions from residential and non-residential sectors. (**d**–**f**) Same as (**a**–**c**) but for CO.

Figure 2 shows time series of the observed and simulated BC and CO levels during Jan–Apr 2020 and 2019 for comparison. The BC mass concentrations at Fukue clearly showed peaks during the transport events from China (green vertical bars on top of the figures). The average BC mass concentration and ΔCO mixing ratio (excess to the baseline, see “Methods”) for Chinese airmasses (108 hourly cases) were 0.471 μg m^−3^ and 96.1 ppb during Feb–Mar 2020, respectively, compared to 0.416 μg m^−3^ and 97.8 ppb, respectively, for the corresponding 166 hourly cases during Feb–Mar 2019 (Fig. 3a, d). The Weather Research and Forecasting (WRF) and Community Multiscale Air Quality (CMAQ) (collectively WRF/CMAQ) model simulations (see “Methods”) reproduced such peaks during the transport events, particularly with respect to the air mass arrival timings and relative magnitudes. Such high performance in simulating the long-range transport events has been established for a long-term period (2009–2019) in our previous study^10^ and herein the simulation was extended to 2020. Note that the model’s performance in Feb–Mar for earlier years (2015–2018) were similarly good (Fig. S3). The simulations confirmed that major increases in the BC and CO levels were associated with air mass transport from China. The absolute levels were not well reproduced by the model, as the emission fluxes were intentionally fixed to those in 2008^16^, which was regarded as a reference year (see details in “Methods”). Though the reference year was somewhat old, the spatial patterns of the emission fluxes were qualified against a newer emission inventory^6^ (Fig. S4), at least with a spatial resolution of province levels, sustaining the performance of the simulations. From the ratios of the observed and modeled levels, we rather aimed to determine emission correction factors, which would bring the model levels into agreement with the hourly observations for the Feb–Mar period of each year^10^. The simulation results were used only when wet removal was regarded negligible, similar to the observations. In the model simulations, the WRF meteorology of the particular time periods were considered, and thus the effect from interannual variability in meteorology, including wind patterns, were removed by evaluating the observation-to-model ratios.

The average BC and ΔCO levels from the model simulations during the selected hours with transport from China without underway wet removal were 1.08 μg m^−3^ and 140 ppb in 2020, respectively, and 0.777 μg m^−3^ and 83.1 ppb in 2019, respectively (Fig. 3a, d). This indicates that wind patterns were favorable for the transport of BC from the continent in 2020 for the analyzed cases. The calculated emission correction factors (median values of the ratios of the individual hours) were 0.55 ± 0.05 and 0.45 ± 0.09 (1σ) for 2019 and 2020 (Fig. 3b), respectively for BC. In this case, the range was estimated from the variability in the median values when the data were categorized into three subgroups (the random subgrouping was done 100 times and the median of the variability was employed). The central values were in good agreement with the observation-to-model ratios (0.54 and 0.44) calculated after averaging concentrations over the annual events for observations and model simulations, respectively. We have previously discussed^10^ that the fact that the values are much less than unity indicates that the BC emission fluxes from China are much lower than the reference value (i.e. 2008 value from REAS version 2.1^16^) and here we reaffirmed the results with a narrower seasonal time window (Feb–Mar). As a further study, we focus on a 18% reduction in the ratios from 2019 to 2020 (i.e., from 0.55 ± 0.05 to 0.45 ± 0.09). In a national-level daily activity change estimation, Forster et al.^12^ estimated a 25% decrease in the Chinese BC emissions during Feb–Mar 2020. Our central estimate of the 18% reduction is smaller, but within the uncertainty range.

We attribute the 18% reduction primarily to the human activity reduction due to the COVID-19 pandemic. The activity levels of the industry and transport sectors (hereafter called non-residential sector collectively) were considerably reduced during this period, while that for the residential sector remained largely unchanged^11,12^. The overall activity in the former category was assumed to be reduced to 0.50 ± 0.14 of the original level, during the two-month period by considering the reported activity reductions^12,17^ (see “Methods”). Considering the reduction, the full contribution from the emission flux arising from the non-residential sector was estimated to be 36%, in 2019 (Fig. 3c), without influence from COVID-19. This indicates that the emission fraction from the uninfluenced sector (i.e., the residential sector) was 64% (50–72%) and dominant (Fig. 3c).

The same analysis for ΔCO yielded median values of the emission correction factors of 1.08 ± 0.08 and 0.70 ± 0.07 (1σ) for 2019 and 2020, respectively (Fig. 3e). The 35% reduction (from 1.08 to 0.70) of CO emission flux in 2020 with respect to 2019 was significantly larger than the 18% estimated for BC in this study and the predicted 26% reduction^12^. Here the major difference in the emitting sectors for BC and CO is suggested.

Assuming that the activity level in the non-residential sector was reduced to 0.50 ± 0.14 of the original level for CO (see “Methods”), the non-residential sectors had a major contribution to the total, by 70% (55–97%), whereas the remaining minor fraction (30%) was attributed to the residential sector (Fig. 3f). This shows a clear contrast with the results for BC. The CO may also be affected by emission changes in the rest of the Northern Hemisphere; however, during this early phase of the COVID-19 prevalence, air masses reflecting emission reductions in Europe or America, where lockdown occurred from middle March 2020, may not have been transported to East Asia yet. Further, such intercontinental transport signals were expected to become broader with time and thus often absorbed in the baseline calculation when estimating ΔCO.

Our results are principally consistent with Cui et al*.*^18^, suggesting the significance of non-traffic source sector from a weak BC emission reduction during the pandemic period in the rural area of Qingdao, Shandong Province, China, in evaluating increases in the BC/NO_2_ concentration ratios. To the contrary, Jia et al*.*^19^ indicated as much as 48–70% reduction in the BC emissions in the northern and eastern China. It should be noted that their evaluation was made on the weekly basis and that they included cases with precipitation, assuming that the FLEXPART model was able to represent the wet deposition. We have previously noticed that the wet deposition efficiency associated with in-cloud scavenging for the most recent FLEXPART version 10.4 was too weak^20^. Considering the similar difficulty in representing wet deposition quantitatively with our WRF/CMAQ model simulations, we avoided using cases with non-negligible influence from wet deposition.

### Evaluation of bottom-up emission inventories

Because of the limited spatial and temporal coverage of the footprint with respect to the distribution of Chinese BC emissions, caution is necessary when our results are compared with the national annual total emissions. Although the important emission regions around central East China (15 provinces or municipalities) were covered by the footprint (Fig. S2), our estimation suggested that the other 46% fraction of the country total BC emissions, particularly those from southern or western areas, might have produced only 2% of the signal at Fukue Island when the synthesized pseudo signal was analyzed by multiplying the footprint with the emission rates. Such non-ideal spatial coverage of the footprint would introduce an uncertainty of ± 10% when comparing the estimated sectoral contributions with those of the whole country. Yet another type of uncertainty (± 10%) is expected with the seasonality, as the analyzed observation period was limited to Feb–Mar. Regional delay or inhomogeneity with the emission reduction could affect our analysis but was regarded negligible as previous studies, as the lockdown policy was introduced uniformly over the country. Thereby the total uncertainty range in the annual residential emission fraction of BC from this study was widened to 44–82% for the central estimate of 64%. Additional uncertainties for CO regarding the limited spatial and temporal coverage were evaluated to be identical; the total uncertainty range in the estimated annual non-residential emission fraction of CO was 48–100% (as a theoretical maximum) for the central estimate of 70%.

Table 1 contrasts the estimated sector fractions with the latest estimates from five bottom-up emission inventories. The fractions from the residential sector for Chinese BC emission fluxes are widely spread among the five inventories, from 22% with EDGAR version 5.0^21^ for 2015 to 63% with ECLIPSE version 6b^22^ for 2015. Our analysis (64% as central value with a range of 44–82%) supports the upper end of the range, particularly from ECLIPSE version 6b. The low residential BC fraction in EDGAR version 5.0 is partly because it attributes a large fraction (33%) to “1.A.1.bc Petroleum Refining – Manufacture of Solid Fuels and Other Energy Industries” in the IPCC categorization, which seems to be too large; this is potentially due to high biased emission factors for coke oven which are expected to have been reduced due to significant transformation of coking sector^23,24^. The non-residential sector fractions for CO from inventories were narrower, ranging from 52 to 75% and were fairly consistent with our central estimate (70%).

**Table 1:** Estimated sectoral emission fluxes of BC and CO from China in 2019, a normal year before the COVID-19 effects.

	BC, residential (Tg yr^−1^)	BC, others (Tg yr^−1^)	CO, residential (Tg yr^−1^)	CO, others (Tg yr^−1^)
This study	0.56 ± 0.23 (64% or 44–82%)	(36%)	(30%)	153 ± 69 (70% or 48–100%)
REAS version 3.2^6^ (2015)	0.67 (40%)	0.99 (60%)	50 (28%)	126 (72%)
MEIC version 1.3^25^ (2017)	0.63 (50%)	0.63 (50%)	57 (42%)	79 (58%)
ECLIPSE version 6b^22^ (2015)	0.74 (63%)	0.43 (37%)	43 (27%)	118 (73%)
EDGAR version 5.0^21^ (2015)	0.29 (22%)	1.03 (78%)	32 (25%)	98 (75%)
CEDS (v_2016_07_16, CMIP6 release)^5^ (2014)	1.29 (51%)	1.25 (49%)	92 (48%)	101 (52%)

For BC, the absolute emission flux from the residential sector was also estimated to be 0.56 ± 0.23 Tg yr^−1^ (Table 1), by multiplying the emission correction factor (0.55 for 2019) associated with an overall uncertainty of ± 27%^10^ and the estimated fraction attributable to residential (64%, or 44–82%) to the original reference BC emission flux (1.59 Tg yr^−1^) assumed in the model simulations. The value was fairly consistent with those from REAS version 3.2^6^, MEIC version 1.3^25^, and ECLIPSE version 6b^22^ (0.63–0.74 Tg yr^−1^). In comparison, our results suggest that EDGAR version 5.0^21^ (2015), had a large underestimation (0.29 Tg yr^−1^), whereas CEDS (CMIP6 release)^5^ had a large overestimation (1.29 Tg yr^−1^). Considering that the bottom-up emission inventories were for 2014–2017, steady emission reductions might have been important from the years until 2019. The total BC emission flux from China, estimated as 0.87 ± 0.27 Tg yr^−1^ for 2019 was on the low side of the range of the 1.17–2.54 Tg yr^−1^, as discussed in our previous study^10^. It should be noted that a newer CEDS version, CEDS_GBD-MAPS (2020_v1.0)^26^ had lower values than 2.54 Tg yr^−1^, i.e., 1.61 and 1.39 Tg yr^−1^ for 2015 and 2017, respectively.

For the sectoral separation, only data from 2019 and 2020 were compared; however, the difference between the two years might have been influenced from a longer-term decreasing trend since 2015 for BC (Fig. 3b); this implies that factors other than the effect of COVID-19 might also have been important. Even so, the decreasing trend in recent years was likely to be driven by the change within the industrial sectors, as no particular measures were evident for the residential sector during this period. In such a case, the BC emission flux decrease from 2019 to 2020 can still be attributable to changes within the industry sector (i.e., non-residential or others) and therefore, our conclusion here that the BC emission flux in 2019 was dominated by the residential sector remains unchanged. For CO, the decrease in the emission correction factor in 2020 was evident, while the factor was almost flat during 2015–2019 (Fig. 3e); the results for CO would also be unchanged even when the data before 2019 were taken into account.

For simplicity we have so far assumed that the emission fluxes from the residential sector were unchanged from 2019 to 2020. However, it has been suggested^12^ that there was a 5% increase in the activity level during Feb–Mar 2020, due to the increased energy demand in the sector related to the stay-home policy. Considering this factor, the contribution from the non-residential sector would be decreased by ~ 2%. Nonetheless the influence is small and will not alter our conclusion that the residential sector was dominant.

For CO, the absolute emission flux from the non-residential sectors estimated from this study, 153 ± 69 Tg yr^−1^, was relatively larger than those from the inventories, in the range of 79–126 Tg yr^−1^. The total CO emission flux from China was estimated as 219 ± 46 Tg yr^−1^, on the high side of the range from the bottom-up emission inventories (136–193 Tg yr^−1^). The cause of the difference has not been identified. It should be noted that a study estimating the Chinese total CO emission flux from satellite data assimilation in a top-down approach^27^ yielded 231.3 Tg yr^−1^ for 2016 for eastern China only.

Another robust parameter determined in this study is the BC/CO emission ratio from the non-residential and residential sector, as 0.0020 gBC gCO^−1^ (or 2.5 ng m^−3^ BC (ppb CO)^−1^ in Standard Temperature and Pressure (STP, 273 K, 1 atm) and 0.0084 gBC gCO^−1^ (or 10.6 ng m^−3^ BC (ppb CO)^−1^ in STP, respectively. The value for the non-residential sector was closest to 0.0036 gBC gCO^−1^ (or 4.6 ng m^−3^ BC (ppb CO)^−1^ in STP) from ECLIPSE version 6b again among the studied bottom-up inventories and much lower than the range from others (0.0079–0.012 gBC gCO^−1^ or (9.8–15.5 ng m^−3^ BC (ppb CO)^−1^ in STP). The latter value for the residential sector was overestimated (0.011–0.017 gBC gCO^−1^ (or 13.8–21.5 ng m^−3^ BC (ppb CO)^−1^ in STP) by the bottom-up inventories, with the closest value from ECLIPSE version 6b. The large spread of the BC/CO emission ratios between the residential and rest sectors by a factor of 4 was found from this study. Except for EDGAR version 5.0, all bottom-up inventories qualitatively estimated a larger emission ratio for the residential sector than that for the non-residential sector, which is consistent with observations. However, the ratios need to be shifted downward considerably to explain the observations.

### Implications for mitigation policy

The BC emission flux from the residential sector, shown to be dominant in 2019 appear as the robust policy target for further emission reduction in China. Its sector-level importance has been highlighted by previous studies^1–4,28^ through quantification of its climate effect or the potential effectiveness of its reduction in terms of health and climate co-benefits. This study provides a clear observational evidence that the dominant Chinese BC emission flux is from the residential sector, which these studies assumed.

**Figure 4:**
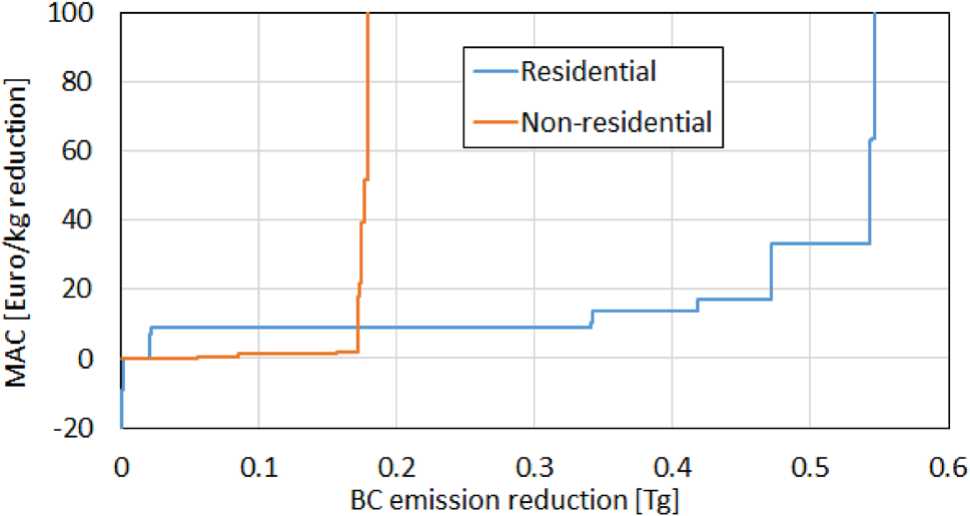
Marginal abatement cost (MAC) curves for Chinese BC emissions from residential and non-residential sectors.

Further in this section we provide analysis of the marginal abatement cost (MAC) curves^29,30^ (Fig. 4, see “Methods”), which can be used to indicate the emission abatement potential and associated abatement costs, and demonstrate economic feasibility for the reduction in residential BC emissions from China. For this analysis, we used detailed information from ECLIPSE version 6b^22^ for 2015, whose performance has been assessed consistent with observations, in terms of the total Chinese BC emission flux as well as its long-term trend in our previous study^10^ and the sector contributions in this study. Figure 4 shows the MAC curves for the residential and non-residential sectors. For the residential sector, the MAC was sustained at around 10–20 Euro kg^−1^ for a BC emission reduction range up to 0.45 Tg, whereas those for the non-residential sector increased to 100 Euro kg^−1^ for a BC reduction < 0.2 Tg. This suggests that the BC emission reduction in the residential sector, mainly achieved by replacing coal-based cooking or heating stoves with gas-based alternatives, is more cost efficient.

## Methods

### Observations and model simulations

The analyzed BC and CO observations were taken from the Fukue atmospheric environment observatory (32.75°N, 128.68°E, 75 m above sea level, see Fig. 1)^10,14,31^. The observatory is located on a remote Fukue Island (326 km^2^) in western Japan, receiving continental outflow airmasses frequently in the winter–spring season, with negligible influence from local emissions on the island (more than 10 km away from the main township). See references^10,14,31^ for more details. Decadal observations of BC since 2009 and CO since 2011 have been conducted, with particular focus on Feb–Mar 2019 and 2020 for this study. The used instruments and their operation were detailed in Kanaya et al.^10^. Briefly, "unified" BC data were produced by averaging hourly observational records from two instruments, a continuous soot-monitoring system (COSMOS; BCM3130, Kanomax, Suita, Japan) and a multi-angle absorption photometer (MAAP5012; Thermo Scientific, Waltham, MA, USA). A PM_1_ cyclone was equipped in the air sampling line. The two instruments are minimally influenced by co-existing scattering particles, because of a pre-heater integration for COSMOS and scattering correction algorithm considered with the MAAP. Using the mass absorption cross section of 10.3 m^2^ g^−1^, the BC mass concentrations from MAAP agreed with those from COSMOS with a systematic high bias of 14% and a random uncertainty of ± 17% for a long observational period (2009–2019)^10^ (See Fig. S2 of the paper for details). For the particular period of this study, a good agreement down to 0.02 μg m^−3^ was found; the high bias of the MAAP and the random uncertainty were estimated as 4% and ± 17% for Feb–Mar during 2015–2019 and 6% and ± 14% for Feb–Mar during 2020, respectively (Fig. S5). The BC levels from COSMOS agreed with those from a Single Particle Soot Photometer (SP2; Droplet Measurement Technologies, Longmont, Colorado, USA) to within 12% in 2015^32^ and 8% in 2019^33^, respectively. The atmospheric CO mixing ratios were observed with a non-dispersive infrared sensor (Model 48C; Thermo Scientific) with an uncertainty of 4%^10,14^. Here, ΔCO was defined as the enhancement from baseline, determined as the 5th percentile over the moving 14-day time window. Hourly airmass classification was made with backward trajectory analysis (NOAA HYSPLIT^13^) from the coordinates of the observation site at an altitude of 500 m above sea level using a meteorological field from the Global Data Assimilation System (GDAS1) at a 1° × 1° resolution and a duration of 120 h, similarly to Kanaya et al*.*^10^. Hourly data measuring air masses directly leaving the coastline of mainland China and arriving at Fukue Island via the East China Sea were identified. Data with minimal influence from wet deposition were selected based on the criterion that Accumulated Precipitation along the Trajectories integrated over the past 72 h (APT^34^) was less than 1 mm, as used by Kanaya et al*.*^10^. Influence of dry deposition was weak, as assessed by the decay of the ΔBC/ΔCO ratio against the air mass travel time^14^. Data during 166 and 108 hourly cases were thus selected for 2019 and 2020, respectively, and were compared. The footprint area, where the observations at Fukue would have sensitivities to surface emissions, was estimated from the spatial distribution of the hourly waypoints of the backward trajectories calculated individually for the 166 and 108 hourly cases (Fig. 1 and Fig. S1). Another type of footprint calculations was also made with the FLEXPART model (version 10.4)^15^ for the same hourly cases (Fig. S2) to verify the results based on the simple backward trajectories, using a meteorological field from the NCEP FNL operational model global tropospheric analyses (ds083.2) at a 1° × 1° resolution. Wet and dry deposition processes were made effective during the transport for 120 h, though considered to be negligible for the selected cases. The two types of footprint maps similarly covered the economy centers of China in CEC, while the footprint with the FLEXPART model extended to a wider region as the air dispersion was represented. The two methodologies agreed in that ~ 50% (specifically 48 or 46%) of BC emissions from China, particularly those from southern or western areas, have produced only 2% of the signal at Fukue Island when the synthesized pseudo signal was analyzed by multiplying the footprint with the emission rates. The 15 provinces or municipalities around CEC producing major signals were identified with the footprint derived with the FLEXPART model, with which the potential representativeness of the estimated sectoral contributions over the country was examined.

Even though cases with negligible effect of wet deposition were chosen, the wind transportation pattern of the outflow was different from case to case or year to year and affects the comparison between 2020 and 2019. This meteorological effect was corrected for by using the Weather Research and Forecasting (WRF) chemical transport model, coupled with the Community Multiscale Air Quality model (CMAQ), collectively WRF/CMAQ. The model was able to reproduce the timings of the events and associated transportation process details, including degrees of horizontal and vertical dispersion specifically for the east Asia region^35–37^. Kanaya et al*.*^10^ demonstrated the superior performance of the model with respect to BC and ΔCO during 2009–2019 in their Figs. 4 and S1. Here, we showed its high performance for 2020 and 2019 (Fig. 2) and 2015–2018 (Fig. S3), which indicates that the spatial distribution of the emission inventory of BC and CO for input to the model (REASv2.1^16^) was reasonable.

The emission fluxes were purposefully kept constant at a reference value (i.e., those estimated for 2008). The emission correction factors were calculated as the observation-to-model concentration ratios on each transport event basis or their two-months mean basis. The annual mean of the ratios could be regarded as factors that bring the model simulations into agreement with observations, assuming a linear response of the concentrations to the emissions. Through this data processing, we removed the effect from interannual variabilities in the transport efficiency and evaluated the effects from the emission changes alone. The method was previously successfully applied for estimating the large emission reduction trend of BC from China^10^ between 2009 and 2019. In this study, the analysis was extended to 2020 by focusing on the events during Feb–Mar, to fit to the two months in 2020 (Fig. 2), under the strong influence from the restricted emissions during the COVID-19 pandemic.

### Estimation of activity level changes

The activity level change in the other (non-residential) sector due to the human activity restriction during Feb–Mar 2020 was estimated as a weighted average of those estimated individually for the surface transport and industry sectors (− 79%^17^ for the surface transport sub-sector and − 33%^12^ for the industry sub-sector during Feb–Mar 2020), as − 50 ± 14% (reduction ratio of 0.50 ± 0.14) for both BC and CO. The values were then used to convert the partial emission reduction, which was directly estimated based on the concentration changes, to the full emission contribution of the non-residential sector. Finally, the full emission contribution of the residential sector was determined based on the remainder. The weights of emissions were estimated by averaging those in the three emission inventories (REAS version 3.2^6^, MEIC version 1.3^25^, and ECLIPSE version 6b^22^). The weights could be different between BC and CO but were assumed to be identical because of the large uncertainties. It should be noted that the estimated activity level change for the surface transport sub-sector was smaller (− 42%)^12^, when estimated based on mobility data, than the − 79%^17^ used here. Future studies based on more solid information, such as car travel distances or fuel use, are needed for validation purposes.

### Calculation of the marginal abatement cost (MAC)

*EF*_*ijk*_ represents the BC emission factor for sub-sector *i*, fuel type *j*, and the applied technology *k*, and *C*_*ijk*_ the cost to apply technology *k* to a unit of fuel *j* in *i*. Assuming that the emission is abated by replacing technologies *h* (high emission) with *l* (low emission), the MAC is calculated as:

1 $$ MAC_{ijhl} = (C_{ijl} {-}C_{ijh} )/(EF_{ijh} {-}EF_{ijl} ). $$

Aggregating *MAC*_*ijhl*_ for all *i, j*, and existing *h*, we obtained the national-level MAC curve and amount of abated BC emissions, while separating the residential and non-residential sectors. A caveat is that the simultaneous emission changes of species other than BC are not included, and the calculation does not intend to cover the effects of all species.

## Supplementary Information

Supplementary Figures.

## Data Availability

The observational data set for BC is collectively available from https://ebcrpa.jamstec.go.jp/atmoscomp/obsdata/ or upon direct contact to the corresponding author.
