# Supplementary material for: Dominance of the residential sector in Chinese black carbon emissions as identified from downwind atmospheric observations during the COVID-19 pandemic
Source: Sci Rep. 2021 Dec 16;11:23378. doi: 10.1038/s41598-021-02518-2 (PMC8677718; doi:10.1038/s41598-021-02518-2)
Supplement: Supplementary file 1 — Supplementary Figures. [file 41598_2021_2518_MOESM1_ESM.docx]

**Dominance of the residential sector in Chinese black carbon emissions as identified from downwind atmospheric observations during the COVID-19 Pandemic**

Yugo Kanaya^1,2,*^, Kazuyo Yamaji^2,1^, Takuma Miyakawa^1^, Fumikazu Taketani^1,2^, Chunmao Zhu^1^, Yongjoo Choi^1^, Kohei Ikeda^3^, Hiroshi Tanimoto^3^, Daichi Yamada^4^, Daiju Narita^5^, Yutaka Kondo^6^, Zbigniew Klimont^7^

^1^Research Institute for Global Change, Japan Agency for Marine-Earth Science and Technology (JAMSTEC), Yokohama, 2360001, Japan

^2^Graduate School of Maritime Sciences, Kobe University, Kobe, 6580002, Japan

^3^Earth System Division, National Institute for Environmental Studies, Tsukuba, 3058506, Japan

^3^Faculty of Economics and Business, Hokkaido University, Sapporo, 0600809, Japan

^5^The University of Tokyo, Tokyo 1538902, Japan

^6^National Institute of Polar Research, Tachikawa, 1908518, Japan

^7^International Institute for Applied Systems Analysis (IIASA), 2361 Laxenburg, Austria

**Corresponding author*: Yugo Kanaya (yugo@jamstec.go.jp)

**Supplementary materials**


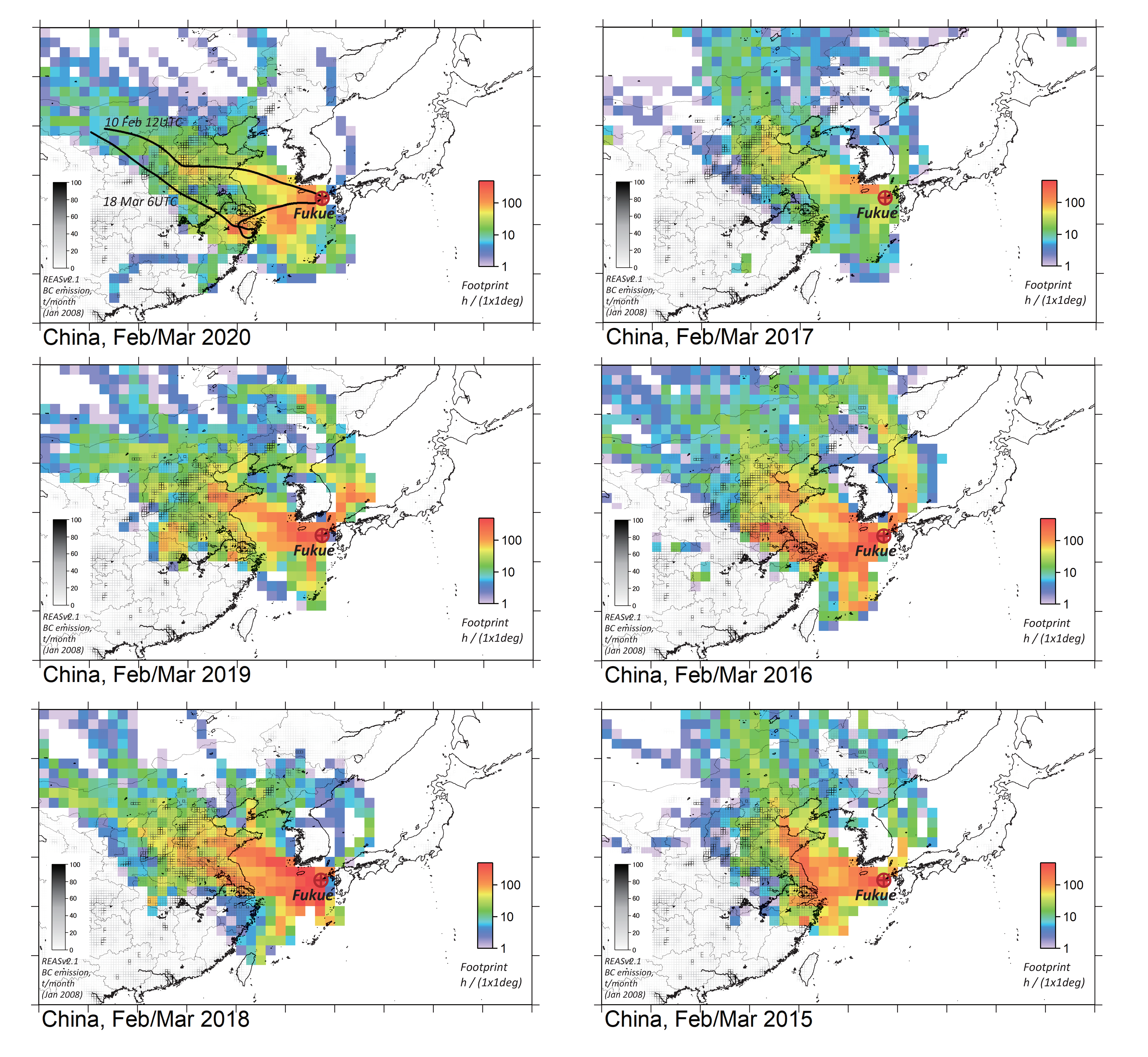


**Figure S1.** Same as Figure 1 but the footprint densities are shown for an extended period during Feb/Mar in 2015–2020.


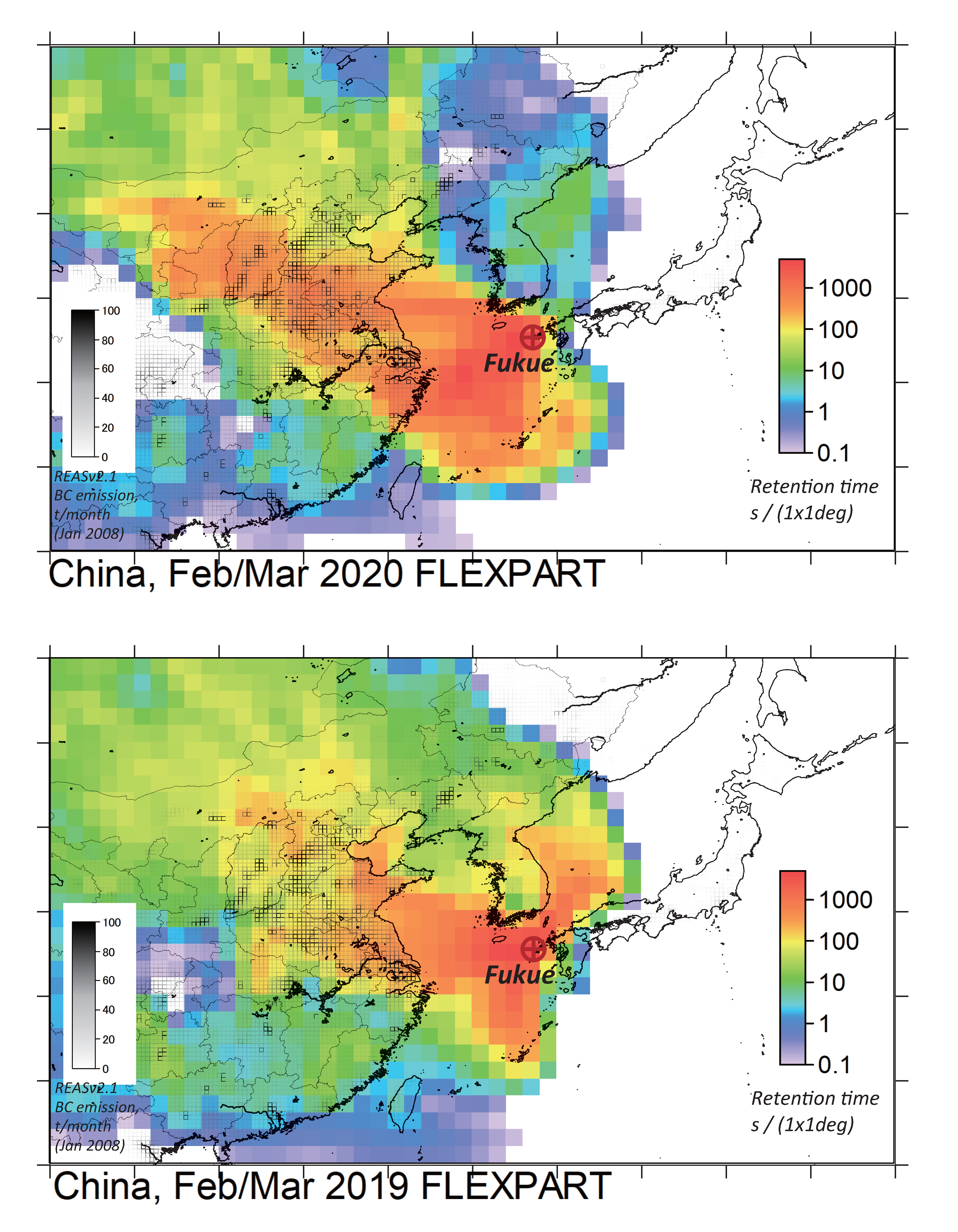


**Figure S2**. Footprint densities calculated with the FLEXPART model during Feb/Mar in 2020 and 2019.


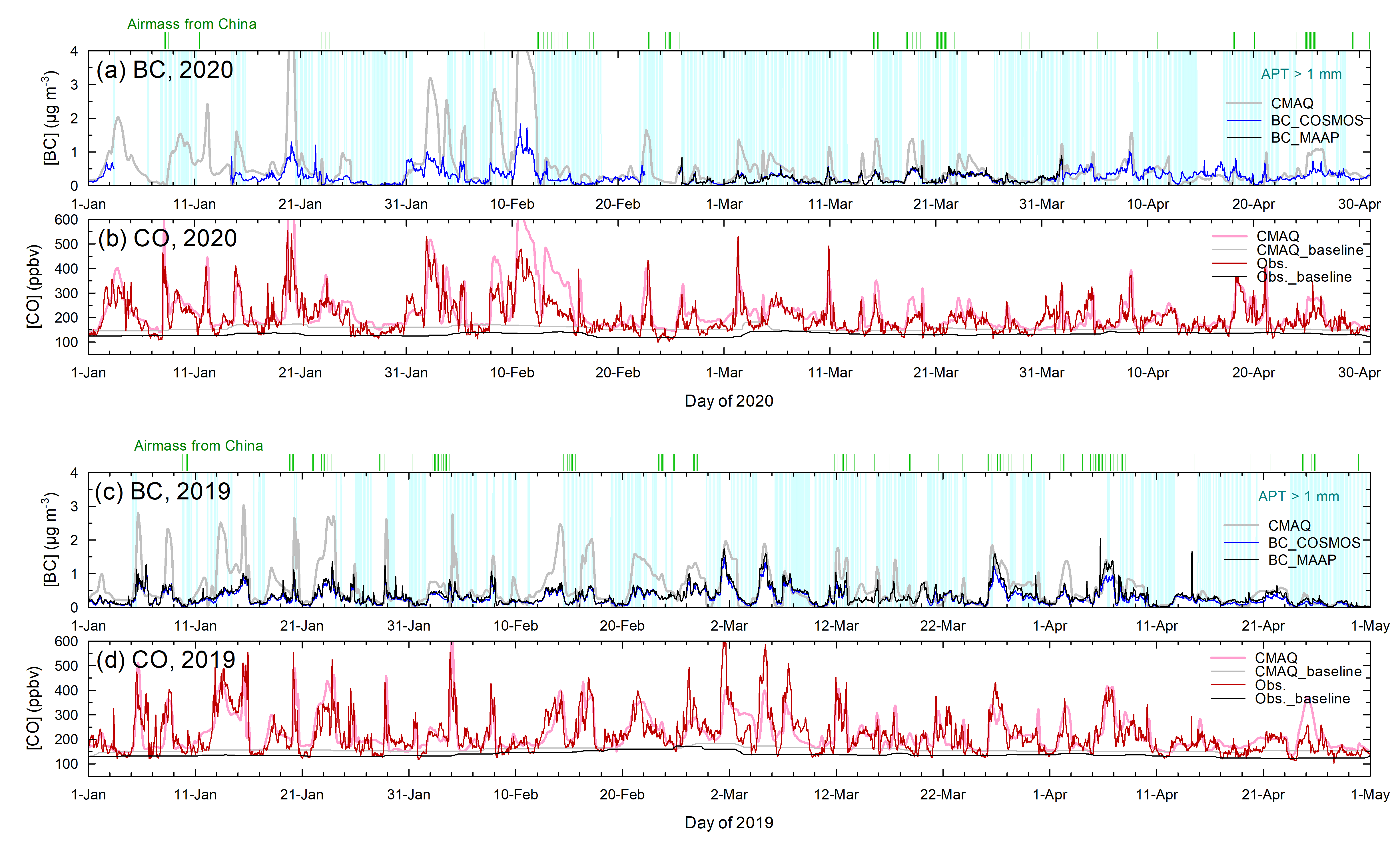

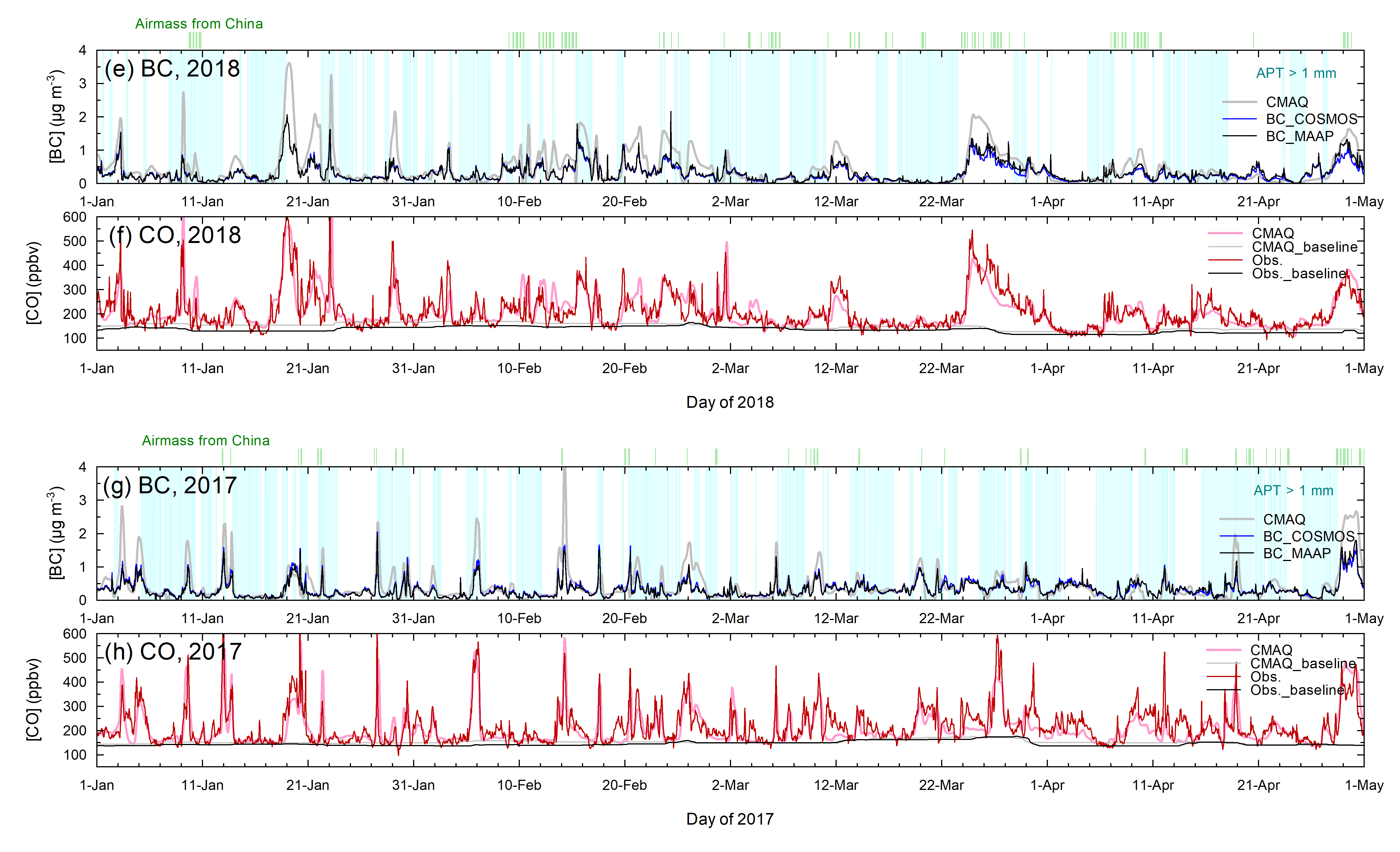


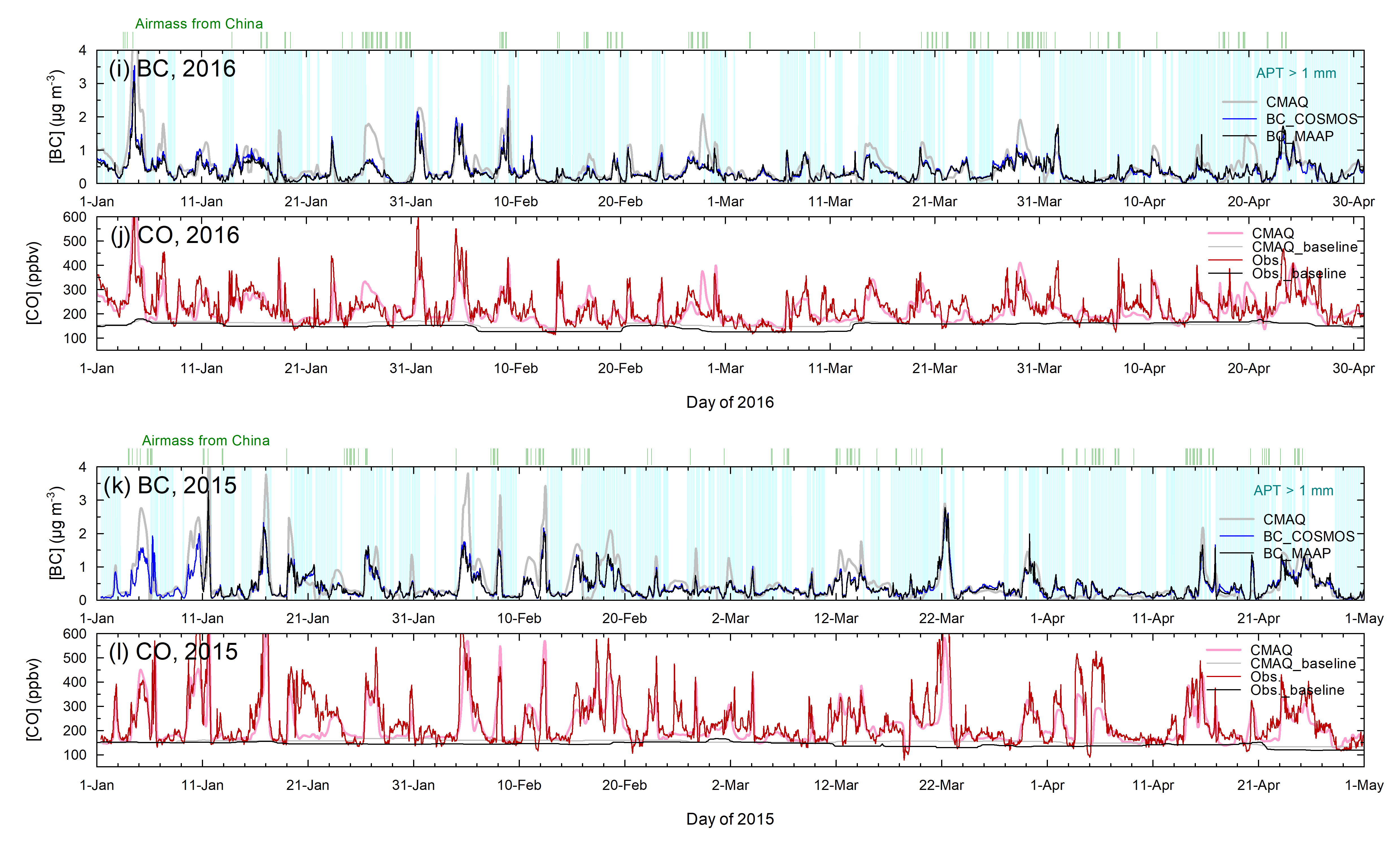


**Figure S3**. Same as Fig.2 but for an extended period during 2015–2020.


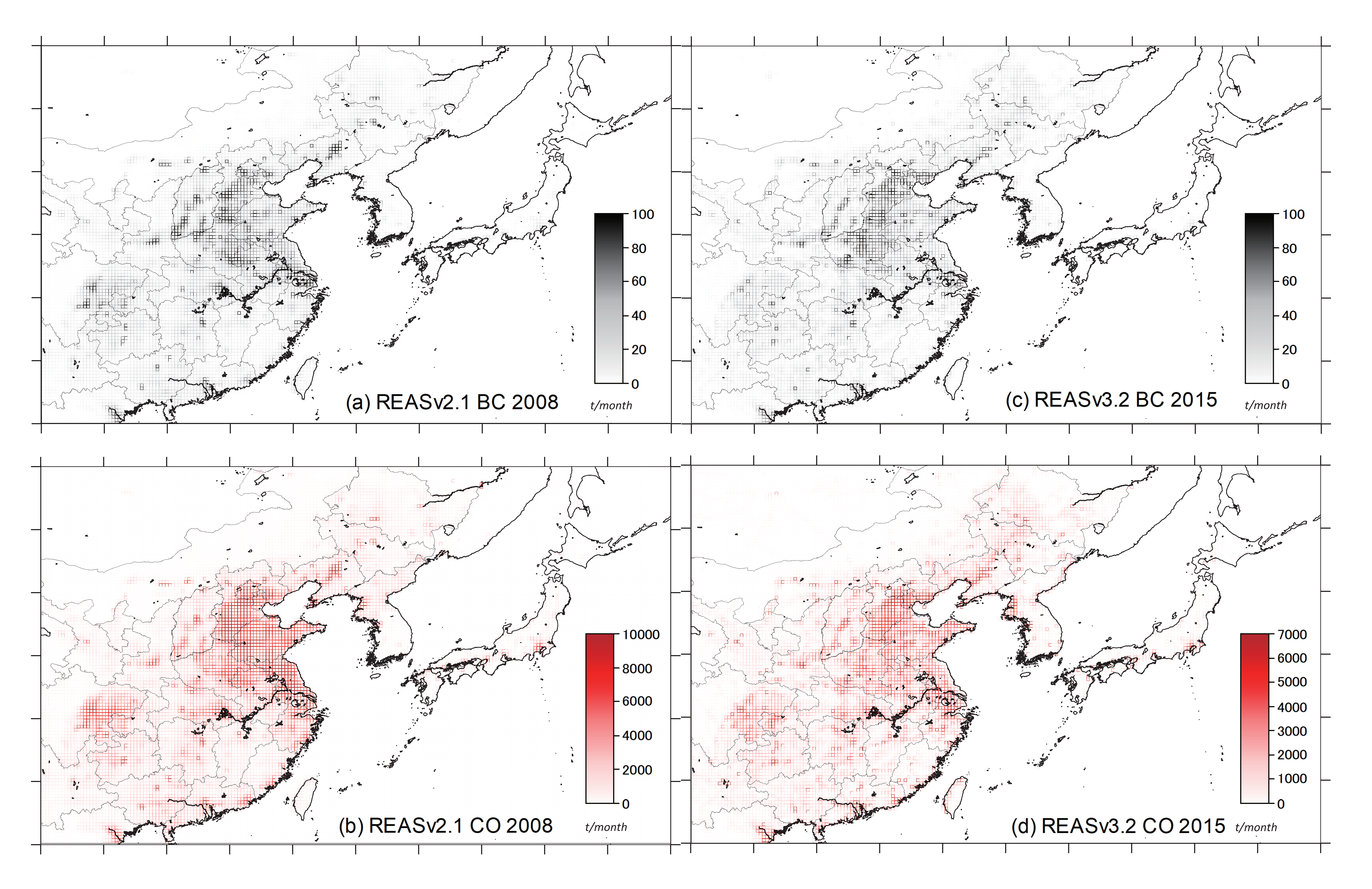


**Figure S4.** Spatial patterns of BC and CO emission inventories from REAS version 2.1 (a and b) for 2008 (used for model input) and from REAS version 3.2 for 2015.


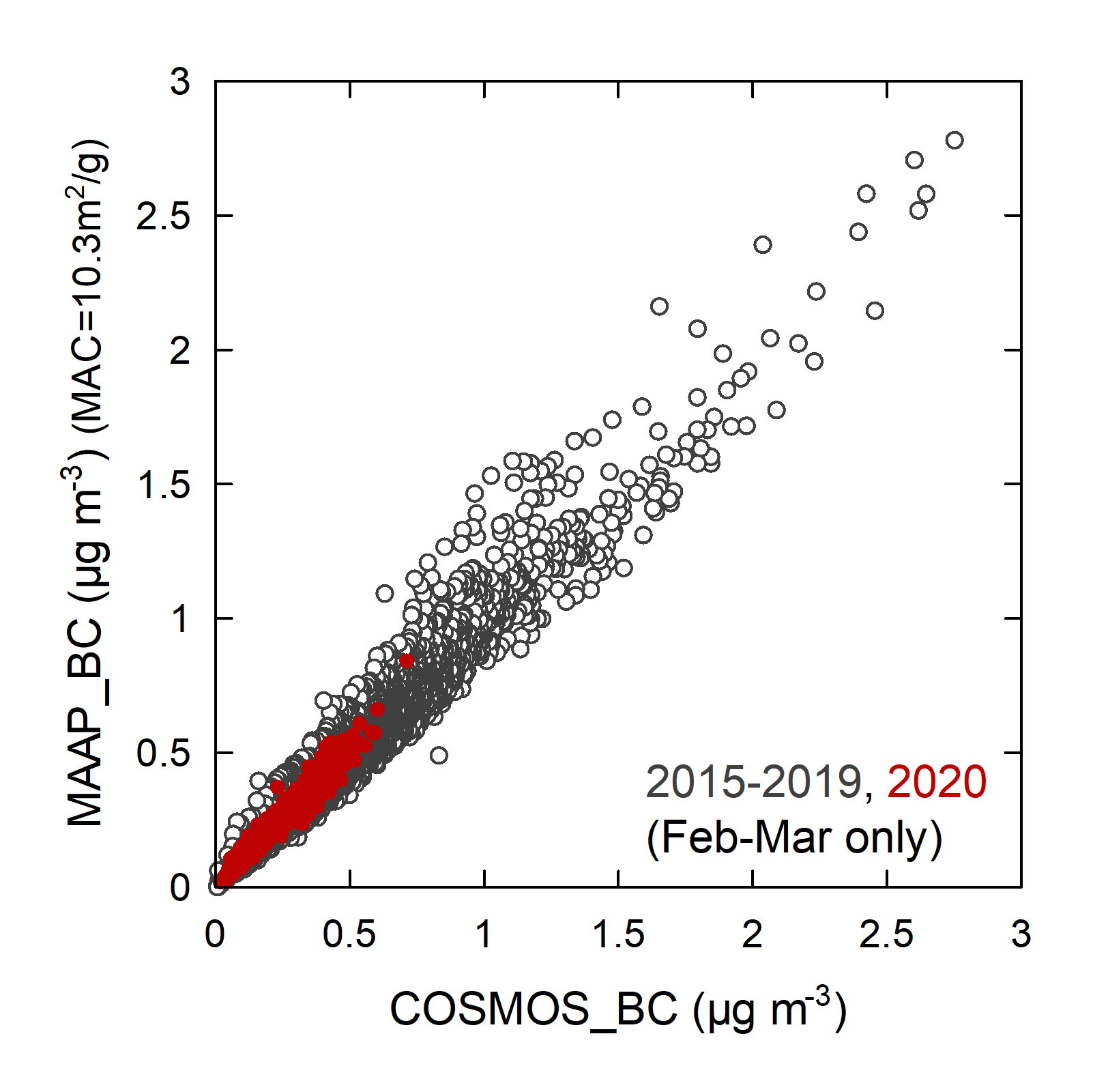


**Figure S5.** Correlation between hourly BC concentrations from COSMOS and MAAP instruments, during Feb–Mar of 2015–2019 (gray) and 2020 (red).
